# Plasma metagenomic cell-free DNA sequencing identifies clinically unsuspected cases of Toxoplasma gondii infection in immunocompromised patients: a single-centre experience

**DOI:** 10.1099/acmi.0.001068.v4

**Published:** 2026-11-12

**Authors:** Lea R. Goren, Alice C. Lehman, Daniel Drozdov, Beth Kristine Thielen

**Affiliations:** 1 University of Minnesota Medical School, Minneapolis, USA; 2 Division of Pediatric Infectious Diseases, University of Minnesota Medical School, Minneapolis, USA; 3 Division of Infectious Diseases and International Medicine, University of Minnesota, Minneapolis, USA; 4 Division of Pediatric Blood and Marrow Transplantation, University of Minnesota, Minneapolis, USA; 5 Division of Stem Cell Transplantation and Children’s Research Center and Pediatric Hematology and Oncology University Children’s Hospital Zurich, University of Zürich, Zürich, Switzerland; 6 Division of Pediatric Hematology and Oncology, Department of Pediatrics, Kantonsspital Aarau, Aarau, Switzerland

**Keywords:** cell-free nucleic acids, haematopoietic stem cell transplantation, high-throughput nucleotide sequencing, immunocompromised host, metagenomics, toxoplasmosis

## Abstract

Toxoplasmosis can be challenging to diagnose, particularly in immunocompromised patients with nonspecific clinical presentations. We present a retrospective, single-centre case series of eight *Toxoplasma gondii* infections identified by plasma metagenomic cell-free DNA (cfDNA) sequencing in allogeneic haematopoietic stem cell transplant recipients. Nested within a larger cohort evaluating cfDNA utilization, this case series highlights a positive clinical impact of cfDNA testing in 5 out of 8 (62.5%) patients, including earlier diagnosis and changes in clinical management. These findings illustrate the potential utility of plasma cfDNA sequencing as a complement to conventional diagnostics across diverse clinical presentations of toxoplasmosis.

## Data summary

No sequence data are included, as these data are retained by Karius and not shared with the submitting medical facility. All specific case data are included in the manuscript. No software or code was generated. The identification of *Toxoplasma* organisms was reported to the ordering clinical facility using a standardized, templated report. Raw sequencing data were not made available to the ordering facility.

## Introduction

*Toxoplasma gondii* is a protozoan parasite that causes widespread human infection, with reported seroprevalence ranging from 11.2 to 45.2% worldwide [1]. The clinical manifestations and consequences of *T. gondii* infection vary substantially according to host characteristics. In immunocompetent individuals, infection is typically mild and self-limited. However, infection during pregnancy, even when asymptomatic or mild in the mother, can have potentially devastating consequences for foetal development. Immunocompromised individuals are at substantially greater risk of severe disease, which may result from either newly acquired infection or reactivation of latent infection [2].

While severe toxoplasmosis classically manifests as central nervous system (CNS) reactivation in patients with acquired immunodeficiency syndrome, it also occurs in solid organ and allogeneic haematopoietic stem cell transplant (HSCT) recipients, patients with malignancies [2] and those receiving immunomodulatory therapy for rheumatic and other inflammatory diseases [3]. Diagnosis is challenging because of the heterogeneous clinical manifestations ranging from undifferentiated fever to localized CNS, pulmonary and cardiac disease to multi-organ failure [4] and substantial clinical overlap with other opportunistic infections. Clinicians are more likely to order *T. gondii* diagnostic testing when patients present with characteristic clinical syndromes, such as ring-enhancing lesions on brain imaging, but may be less likely to consider toxoplasmosis in patients with nonspecific clinical presentations.

Diagnosis of toxoplasmosis is further complicated by limitations of available diagnostic tests. No single test serves as a gold standard across all clinical manifestations of toxoplasmosis, and the sensitivity and specificity of individual tests vary by disease presentation and host characteristics [5]. PCR can detect low concentrations of *T. gondii* DNA in blood, cerebrospinal fluid (CSF) and other clinical specimens. However, its utility may be limited by the need for invasive sampling and delays in obtaining results when specimens must be shipped to reference laboratories. Serologic testing may be falsely negative in immunocompromised patients and during early infection, sometimes requiring convalescent titres to confirm infection. Immunoglobulin replacement therapy may further compromise the interpretation of serologic testing, particularly in immunocompromised patients. Finally, detection of *T. gondii*–specific IgG does not distinguish inactive latent infection from reactivation in seropositive patients.

Although plasma cell-free DNA (cfDNA) sequencing has limitations in routine clinical practice [6], it may improve diagnostic yield in specific clinical settings where conventional testing is limited, such as acute Q fever [7]. Such testing may also reduce the risks associated with invasive sampling of difficult-to-access anatomic sites. For these reasons, we hypothesized that cfDNA testing would increase the diagnostic yield for toxoplasmosis in immunocompromised hosts, particularly when toxoplasmosis was not strongly suspected based on the clinical presentation. To test this hypothesis, we reviewed all cases of cfDNA testing performed at our institution [8] and identified eight cases of *T. gondii* infection from this larger series. Our primary objective was to describe the clinical characteristics and diagnostic impact of cfDNA sequencing in these cases, rather than to formally estimate the assay’s sensitivity or specificity. We also identify priorities for future research, including systematic head-to-head comparisons with conventional diagnostic modalities and evaluation of how cfDNA testing could be incorporated into diagnostic algorithms.

## Methods

For quality improvement purposes, electronic health records for all patients at the University of Minnesota Medical Center (UMMC) who underwent at least one cfDNA sequencing test (Karius^®^, Redwood City, California) were independently reviewed by two reviewers, including at least one practising infectious diseases physician, as previously described [8]. We extracted relevant clinical data, including patient demographics, underlying conditions, indications for testing, conventional microbiological results and time to diagnosis, into a REDCap database. We then performed a subgroup analysis of patients in whom *T. gondii* was identified. Changes in clinical management were defined according to criteria proposed by Hogan *et al*. [6].

All testing was ordered at the discretion of the treating clinician(s), and no institutional testing guidance or stewardship criteria were in place during the study period. Whole-blood samples were collected in either BD Vacutainer plasma preparation tubes or K2-EDTA tubes. Plasma was separated from cells and stored at ambient temperature for up to 96 h or at −20 °C for up to 6 months before shipment to the Karius, Inc. clinical laboratory (Redwood City, CA), which is certified under the Clinical Laboratory Improvement Amendments of 1988 (CLIA) and accredited by the College of American Pathologists (CAP) for plasma microbial cell-free DNA (mcfDNA) sequencing. mcfDNA sequencing was performed as previously described [9, 10]. Sequencing data were analysed using Karius Test bioinformatic pipeline versions DC-3.1 through DC-3.16, which detect mcfDNA from more than 1,000 microbial taxa and quantify the absolute plasma concentration of mcfDNA in molecules per microlitre (MPM). Because MPM values are not directly comparable across microbial taxa, clinical reports provide a reference interval derived from a study of 675 asymptomatic adults to aid interpretation of individual results.

## Results

A total of 1,331 unique plasma cfDNA tests were performed at UMMC during the study period (December 2017 to March 2024), of which 8 (0.6%) tests detected *T. gondii* DNA (Table 1). Among these eight patients, the most common indication for cfDNA testing was fever with respiratory symptoms and/or pulmonary nodules (6/8), followed by isolated fever (1/8) and headache without fever (1/8). The median time from hospitalization to reporting of cfDNA sequencing results was 9.5 days (IQR 8–18.5), with a median assay turnaround time of 3 days, compared with same-day turnaround for *Toxoplasma* IgG and 1–5 days for *Toxoplasma* PCR. The median *T. gondii* concentration reported was 5,081 MPM for *T. gondii* (IQR 66–12,767). The median patient age was 38 years (IQR 17–62). All patients had a history of allogeneic HSCT; transplant characteristics are detailed in Table 2. The median interval from allogeneic HSCT to cfDNA result reporting was 51 days (IQR 40–53), with all but one patient undergoing cfDNA sequencing within 2 months of transplantation. Of the seven patients receiving *Pneumocystis jirovecii* prophylaxis at the time of diagnosis, none were receiving prophylaxis with activity against *T. gondii*; however, two had received trimethoprim-sulfamethoxazole (TMP-SMX) earlier in their post-transplant course.

**Table 1.** Diagnostic characteristics and clinical impact associated with positive plasma cfDNA testing for *T. gondii*

Case no.	Gender	Age (5-year range)	Initial presentation	Time from HSCT to cfDNA report (days)	cfDNA results (MPM)	Conventional *T. gondii* testing	Infectious disease diagnoses	Clinical impact of cfDNA testing
1	M	20–24	Fevers, new nodular left forearm lesion, new pulmonary nodules	19	*T. gondii* 16; human herpesvirus 6B 1657	Skin biopsy immunohistochemical stain positive; serum PCR negative, serum IgG positive; CNS lesions present on head imaging; CSF PCR negative	Cutaneous toxoplasmosis, *Staphylococcus epidermidis* bacteraemia	Earlier diagnosis
2	F	65–69	Fevers, new pulmonary nodules	30	*T. gondii* 929; CMV 740; BK virus 58	Serum PCR negative; serum IgG positive; CNS lesions absent on head imaging	Toxoplasmosis, CMV viraemia	Earlier diagnosis, avoided invasive testing, earlier treatment initiation, appropriate treatment de-escalation
3	F	55–59	Fevers, acute hypoxic respiratory failure	51	*T. gondii* (non-quantifiable); *Rhizopus delemar* (non-quantifiable)	Patient died prior to obtaining conventional testing	Diagnosed with disseminated Zygomycetes infection and haemophagocytic lymphohistiocytosis prior to return of cfDNA testing	Patient died prior to return of cfDNA testing
4	M	35–39	Fevers, cough, new pulmonary nodules, diarrhoea	51	*T. gondii* 6217; CMV 3354	Serum PCR positive; serum IgG positive; BAL PCR positive; CNS lesions absent on head imaging	Pulmonary toxoplasmosis, CMV viraemia, BK viraemia, methicillin-resistant *Staphylococcus epidermidis* bacteraemia	Earlier diagnosis
5	M	10–14	Headache	309	*T. gondii* 5081; *Enterococcus faecium* 1698; CMV 624; *Lactobacillus rhamnosus* 542; *Saccharomyces cerevisiae* 372; *Rothia mucilaginosa* 176	Serum IgG negative; CNS lesions present on head imaging; CSF PCR positive	CNS toxoplasmosis	Confirmed diagnosis, appropriate treatment de-escalation
6	F	40–44	Fevers, acute cough, new pulmonary nodules, dysuria	52	*T. gondii* 75328; CMV 940	Serum PCR positive; serum IgG positive; CNS lesions absent on head imaging; CSF PCR positive	Toxoplasmosis, low-grade CMV viraemia, BK viruria	Earlier diagnosis, earlier treatment initiation
7	F	65–69	Fevers, shortness of breath, bilateral pleural effusions, pericardial effusion	50	*T. gondii* 12767; BK virus 703	Serum PCR positive; serum IgG (pre-transplant) positive; CNS lesions absent on head imaging; pleural fluid PCR and cytology negative	Toxoplasmosis, BK viruria	Confirmed diagnosis
8	M	10–14	Fever	54	*T. gondii* 66	Serum PCR positive; serum IgG (pre-transplant) positive; CNS lesions present on head imaging; CSF PCR negative	Toxoplasmosis	Confirmed diagnosis

Organisms are listed as non-quantifiable if they were detected above statistical thresholds but did not meet the minimum sequencing coverage metric.

CMV, cytomegalovirus.

**Table 2.** Patient characteristics associated with positive plasma cfDNA testing for *T. gondii*

Case no.	Gender	Age (5-year range)	Indication for HSCT	Stem cell source	Conditioning regimen	GvHD prophylaxis	*Pneumocystis* ppx	*Toxoplasma* ppx	Outcome
1	M	20–24	High-risk CNS-negative AML	MMUD UCB	Fludarabine, cyclophosphamide, TBI	Tacrolimus, MMF	Pentamidine due to sulfa allergy	None	Died day +156 from relapsed disease
2	F	65–69	AML	MUD BM	Fludarabine, cyclophosphamide, ATG	Tacrolimus, MMF	None (onset of disease prior to scheduled start on day +28)	None	Alive
3	F	55–59	AML	MMUD UCB	Fludarabine, cyclophosphamide, TBI, ATG	Tacrolimus, MMF	TMP-SMX switched to pentamidine due to acute kidney injury	TMP-SMX stopped 3 weeks prior to diagnosis	Died day +48 from disseminated Zygomycetes infection
4	M	35–39	B-cell ALL	MMUD UCB	Fludarabine, cyclophosphamide, TBI	Tacrolimus, MMF	Pentamidine	None	Died day +128 from multiple viral infections
5	M	10–14	Aplastic anaemia	MUD BM	Fludarabine, cyclophosphamide, ATG, TBI	Cyclosporine, MMF	Pentamidine due to neutropenia	None	Alive
6	F	40–44	AML	MMUD double UCB	MAC (fludarabine, cyclophosphamide, TBI)	Tacrolimus, MMF	Pentamidine due to cytopenia	None	Alive
7	F	65–59	t-MDS	MUD PBSC	Fludarabine, cyclophosphamide, TBI	Post-transplant cyclophosphamide, sirolimus, MMF	TMP-SMX switched to pentamidine due to cytopenia	TMP-SMX stopped 3 weeks prior to diagnosis	Alive
8	M	10–14	MDS	1: haploidentical paternal alpha/beta-depleted PBSC (primary graft failure), haploidentical paternal fresh marrow	1: busulfan, cyclophosphamide, rituximab; 2: fludarabine, ATG, cyclophosphamide	1: alpha/beta T cell depletion; 2: post-transplant cyclophosphamide, tacrolimus, MMF	Pentamidine	Atovaquone started but stopped 6 weeks prior to diagnosis due to GI intolerance	Alive

ALL, acute lymphoblastic leukaemia; AML, acute myeloid leukemia; ATG, anti-thymocyte globulin; BM, bone marrow; MMF, mycophenolate mofetil; MMUD, mismatched unrelated donor; MUD, matched unrelated donor; PBSC, peripheral blood stem cell; Ppx, prophylaxis; TBI, total body irradiation; (t-)MDS, (therapy-related) myelodysplastic syndrome; UCB, umbilical cord blood transplant.

Among patients who underwent serological testing, 86% (6/7) had positive *T. gondii* IgG, while serum *T. gondii* PCR was positive in 67% (4/6). CNS lesions on magnetic resonance imaging were present in 43% (3/7) of patients. Of the four patients who underwent lumbar puncture, two (50%) had positive *T. gondii* CSF PCR. One patient had pulmonary toxoplasmosis confirmed by PCR-positive bronchoalveolar lavage (BAL), while five others had chest imaging findings consistent with possible pulmonary toxoplasmosis but lacked invasive confirmatory testing. One patient with pulmonary nodules also developed a skin nodule that was confirmed as cutaneous toxoplasmosis by immunohistochemistry.

Overall, cfDNA testing had a positive clinical impact in 62.5% (5/8) of patients in this selected cohort with positive cfDNA results. Clinical impacts included earlier diagnosis of *T. gondii* infection in 50% (4/8), avoidance of invasive diagnostic procedures in 12.5% (1/8), earlier initiation of appropriate therapy in 25% (2/8) and appropriate de-escalation or discontinuation of unnecessary antimicrobial therapy in 25% (2/8). cfDNA testing had no clinical impact in 37.5% (3/8) of patients because one patient died before cfDNA results became available, and in two patients, conventional testing established the diagnosis before cfDNA results were reported. Excluding the patient who died before diagnosis, all seven patients diagnosed with toxoplasmosis received targeted therapy.

## Discussion

In this cohort of HSCT recipients with toxoplasmosis, cfDNA testing had a positive clinical impact in 62.5% (5/8) of patients, although these findings should be interpreted cautiously given the small sample size and lack of a control group. Positive clinical impacts included earlier diagnosis, avoidance of invasive diagnostic procedures (BAL or lumbar puncture), earlier initiation of appropriate antimicrobial therapy and earlier de-escalation or discontinuation of unnecessary antimicrobials. Despite positive cfDNA results for *T. gondii*, four patients underwent confirmatory invasive testing, potentially reflecting uncertainty regarding the clinical interpretation of cfDNA results.

Although the specificity of cfDNA testing has been questioned for some organisms, our findings suggest that detection of *T. gondii* cfDNA in symptomatic HSCT recipients should prompt consideration of active toxoplasmosis rather than be presumed to represent contamination, particularly when supported by compatible clinical findings or conventional diagnostic testing. In our series, seven of eight patients had additional evidence supporting clinically significant infection: six had confirmation by a direct diagnostic method (PCR or immunohistochemistry), while one had positive serology and a clinical response to treatment. These findings increase confidence that cfDNA detection represented clinically relevant infection rather than contamination. Our observations add to a growing body of case reports demonstrating the potential utility of cfDNA sequencing for diagnosing toxoplasmosis [11–13].

Our study highlights the importance of considering and testing for *T. gondii* in allogeneic HSCT recipients, particularly during the early post-transplant period. Seven of eight cases occurred within 100 days of allogeneic HSCT, raising the possibility that these infections represented reactivation of latent toxoplasmosis in the setting of immunosuppression. Prophylaxis against *P. jirovecii* with TMP-SMX typically begins ~28 days after HSCT; however, pentamidine or atovaquone may be used as alternatives and do not provide adequate protection against *T. gondii* [14]. A recent retrospective study of allogeneic HSCT recipients who were seropositive for *T. gondii* found that a significantly higher proportion of patients with toxoplasma reactivation had not received anti-*T. gondii* prophylaxis (91% vs. 48%) [15]. The study also found that TMP-SMX prophylaxis was associated with improved survival and reduced non-relapse mortality. Together with our findings, these data support consideration of pre-HSCT *T. gondii* serological screening and appropriate anti-*T. gondii* prophylaxis in seropositive recipients [14, 16].

Our cohort consisted exclusively of HSCT recipients, which may be partially explained by the relatively high use of cfDNA sequencing among HSCT patients at our institution (44%, 545/1,331) [8]. Further studies are needed to evaluate the utility of cfDNA sequencing for identifying *T. gondii* infection in other immunocompromised populations at risk for reactivation and severe disease. Given the heterogeneous clinical presentations observed in our cohort, unbiased metagenomic testing may complement conventional targeted diagnostics across a broader range of clinical syndromes in patients at risk for toxoplasmosis, particularly those who are seropositive.

Two interrelated limitations of our study are its retrospective design and the inconsistent use of reference testing to establish the diagnosis. Because cases were identified retrospectively, the selection and timing of conventional diagnostic tests were at the discretion of the treating physician(s) and were not standardized across patients. Furthermore, because our review was limited to patients with positive cfDNA results, we lacked a comparison group of patients diagnosed through conventional *T. gondii* testing. Thus, the sensitivity of cfDNA sequencing for toxoplasmosis cannot be determined from this study. A prospective, multicentre study systematically comparing standard-of-care diagnostic testing with plasma cfDNA sequencing will be important to establish the diagnostic performance of cfDNA and inform an optimal diagnostic algorithm for *T. gondii* infection in immunocompromised patients.

## Conclusion

In this case series of eight HSCT recipients with toxoplasmosis identified by cfDNA sequencing, cfDNA testing had a positive clinical impact in 62.5% of cases, suggesting potential utility when clinical presentations are nonspecific, invasive diagnostic procedures carry substantial risk or conventional testing has limitations. These findings warrant validation in larger, prospective cohorts.

## Abbreviations

BAL: bronchoalveolar lavage
cfDNA: cell-free DNA
CNS: central nervous system
CSF: cerebrospinal fluid
HSCT: haematopoietic stem cell transplant
mcfDNA: microbial cell-free DNA
MPM: molecules per microlitre
TMP-SMX: trimethoprim-sulfamethoxazole
UMMC: University of Minnesota Medical Center
